# Feasibility and safety of left bundle branch area pacing for patients with stable coronary artery disease

**DOI:** 10.3389/fcvm.2023.1246846

**Published:** 2023-11-30

**Authors:** Yu Shan, Maoning Lin, Xia Sheng, Jiefang Zhang, Yaxun Sun, Guosheng Fu, Min Wang

**Affiliations:** ^1^ Department of Cardiology, Sir Run Run Shaw Hospital, College of Medicine, Zhejiang University, Hangzhou, China; ^2^ Key Laboratory of Cardiovascular Intervention and Regenerative Medicine of Zhejiang Province, Hangzhou, China

**Keywords:** left bundle branch area pacing (LBBaP), stable coronary artery disease, pacing parameters, heart failure hospitalization (HFH), safety

## Abstract

**Aims:**

Stable coronary artery disease (CAD) is a prevalent comorbidity among patients requiring pacemaker implantation. This comorbidity may have an impact on the safety and prognosis of traditional right ventricular pacing (RVP). Left bundle branch area pacing (LBBaP) is a new physiological pacing modality. Our aim was to investigate the feasibility and safety of LBBaP in patients with the stable CAD.

**Methods:**

This study included 309 patients with symptomatic bradycardia who underwent LBBaP from September 2017 to October 2021. We included 104 patients with stable CAD (CAD group) and 205 patients without CAD (non-CAD group). Additionally, 153 stable CAD patients underwent RVP, and 64 stable CAD patients underwent His-bundle pacing (HBP) were also enrolled in this study. The safety and prognosis of LBBaP was assessed by comparing pacing parameters, procedure-related complications, and clinical events.

**Results:**

During a follow-up period of 17.4 ± 5.3 months, the safety assessment revealed that the overall rates of procedure-related complications were similar between the stable CAD group and the non-CAD group (7.7% vs. 3.9%). Likewise, similar rates of heart failure hospitalization (HFH) (4.8% vs. 3.4%, stable CAD vs. non-CAD) and the primary composite outcome including death due to cardiovascular disease, HFH, or the necessity for upgrading to biventricular pacing (6.7% vs. 3.9%, stable CAD vs. non-CAD), were observed. In stable CAD patients, LBBaP demonstrated lower pacing thresholds and higher R wave amplitudes when compared to HBP. Additionally, LBBaP also had significantly lower occurrences of the primary composite outcome (6.7% vs. 19.6%, *P* = 0.003) and HFH (4.8% vs. 13.1%, *P* = 0.031) than RVP in stable CAD patients, particularly among patients with the higher ventricular pacing (VP) burden (>20% and >40%).

**Conclusion:**

Compared with non-CAD patients, LBBaP was found to be attainable in stable CAD patients and exhibited comparable mid-term safety and prognosis. Furthermore, in the stable CAD population, LBBaP has demonstrated more stable pacing parameters than HBP, and better prognostic outcomes compared to RVP.

## Introduction

Right ventricular pacing (RVP) engenders cardiac desynchrony and is correlated with a heightened prevalence of left ventricular dysfunction, pacing-induced cardiomyopathy, and mortality (1–3). Compared with RVP, biventricular pacing (BVP) has the potential to mitigate left ventricular mechanical desynchrony and reduction of left ventricular ejection fraction (LVEF) in bradycardia patients (4, 5). However, previous studies have found that BVP is not suitable for routine treatment of bradycardia patients due to its procedural complexity and high cost (6, 7).

In contrast, extensive research has found that permanent His-bundle pacing (HBP) is a more physiological alternative to RVP, exhibiting superior clinical outcomes in comparison to RVP (8, 9). Furthermore, HBP has the capability to rectify left bundle branch block (LBBB) and holds the promise of delivering more effective ventricular resynchronization compared to BVP (10, 11). However, the widespread adoption of HBP has been limited due to challenges such as a steep learning curve, reduced R wave amplitude, and an observed trend of pacing threshold elevation over time associated with the possibility of loss of capture (12, 13).

Left bundle branch area pacing (LBBaP), originally described in 2017 (14), has gained greater acceptance in recent years due to the similar normal paced QRS duration (QRSd), more stable pacing thresholds, better R wave amplitudes which results in better sensing of ventricular activation compared to HBP. Based on the existing body of evidence, it appears that LBBaP represents a viable and efficacious substitute for conventional pacing modalities (15–17).

Coronary artery disease (CAD) is acknowledged globally as one of the foremost causes of disease burden, affecting millions of individuals worldwide (18). As a prevalent comorbidity among patients with pacemaker implantation, CAD might exert an influence on the safety and prognosis of patients with permanent pacemaker implantation (19, 20). Considering that the majority of CAD patients undergoing coronary angiography (CAG) and pacemaker implantation during the same hospitalization are in a stable condition, investigating the impact of stable CAD on pacemaker implantation patients has potentially important clinical implications.

Compared to non-CAD, stable CAD has distinctive features, including myocardial ischemia, and an elevated susceptibility to bleeding associated with the administration of antiplatelet agents. These disparities may amplify the inherent risks associated with the implantation procedure, leading to potential complications such as pocket hematoma and pocket infection (21, 22). Additionally, comorbidities like diabetes and cerebrovascular disease are widespread among individuals with stable CAD, potentially exacerbating the prognosis associated with cardiac pacemaker implantation (23).

While LBBaP has attracted significant attention in recent years due to its stable pacing parameters and the improvement of left ventricular mechanical function, severe myocardial ischemia may impact pacing parameters and prognosis (20). Moreover, the inevitable transseptal lead fixation may increase the risks of implantation complications. To our current understanding, there has been a dearth of studies dedicated to examining the feasibility and safety of LBBaP in individuals with stable CAD. Consequently, the objective of this observational study was to assess the feasibility and safety of LBBaP in patients afflicted with stable CAD compared to those with non-CAD. In addition, this study also encompassed patients diagnosed with stable CAD who had previously undergone RVP or HBP and compared them to stable CAD patients who received LBBaP.

## Methods

### Study population

**Figure 1:**
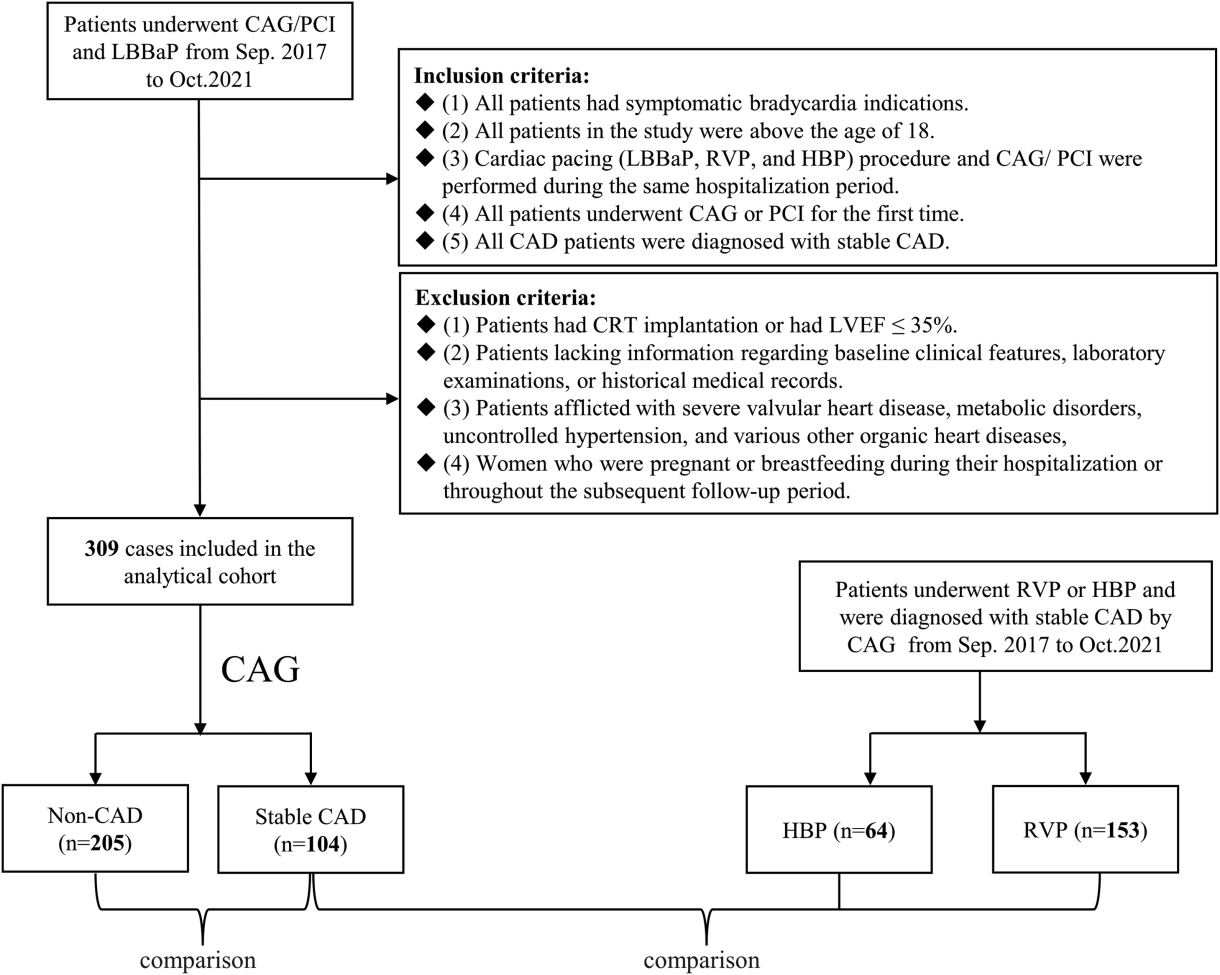
Flowchart of patient enrollment. CAG, coronary angiography; PCI, percutaneous coronary intervention; LBBaP, left bundle branch area pacing; RVP, right ventricular pacing; HBP, his-bundle pacing; CAD, coronary artery disease; CRT, cardiac resynchronization therapy; LVEF, left ventricular ejection fraction.

This retrospective study enrolled 309 consecutive patients who underwent LBBaP and either CAG or percutaneous coronary intervention (PCI) between September 2017 to October 2021 at Sir Run Run Shaw Hospital. They were subsequently categorized into two distinct groups derived from the results of CAG: a stable CAD group and a non-CAD group. An additional cohort of 217 patients, all diagnosed with stable CAD, was also included in the study. Among these patients, 153 underwent RVP (referred to as the RVP group), and 64 underwent HBP (referred to as the HBP group), see Figure 1. All the patients mentioned above conformed to the following inclusion and exclusion criteria.

The study's inclusion criteria were:

(1) all patients had symptomatic bradycardia,
(2) all patients in the study were above the age of 18,
(3) cardiac pacing (LBBaP, RVP, and HBP) procedure and CAG/PCI were performed during the same hospitalization period,
(4) all patients underwent CAG or PCI for the first time,
(5) all CAD patients were diagnosed with stable CAD.

The exclusion criteria were:

(1) patients had cardiac resynchronization therapy (CRT) implantation or had LVEF ≤ 35%,
(2) patients lacking information regarding baseline clinical features, laboratory examinations, or historical medical records,
(3) patients afflicted with severe valvular heart disease, metabolic disorders, uncontrolled hypertension, and various other organic heart diseases,
(4) women who were pregnant or breastfeeding during their hospitalization or throughout the subsequent follow-up period.

The research protocol received approval from the institutional review board of the hospital. The Medical Ethical Review Committee of Sir Run Run Shaw Hospital granted ethical approval for the study, under the reference number 20210420–12.

### Definitions

According to the 2021 ESC guidelines, the definition of “symptomatic bradycardia” encompasses ECG-recorded manifestations such as sick sinus syndrome (SSS), atrial fibrillation (AF) characterized by a prolonged R-R interval, or atrioventricular block (AVB) (24).

A diseased vessel was defined as the presence of a stenotic lesion of ≥50% based on visual angiographic assessment. The diseased vessel was determined by assessing involvement of the epicardial segments of the four major arteries: the left main coronary artery (LM), left anterior descending artery (LAD), left circumflex artery (LCX), and right coronary artery (RCA). Multivessel disease was deemed present in patients with two or more diseased vessels.

### Cardiac pacing procedure (LBBaP, RVP, and HBP)

**Figure 2:**
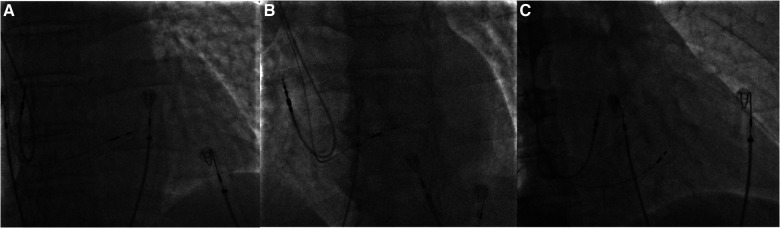
(**A–C**) The 3,830 lead reached the area of LBB in the same patient during procedure. (**A**) The 3,830 lead in the AP position. (**B**) The 3,830 lead in the LAO position. (**C**) The 3,830 lead in the RAO position. LBB, left bundle branch; AP, anteroposterior; LBB, left bundle branch; LAO, left anterior oblique; RAO, right anterior oblique.

Each patient received standard medical treatment tailored to their individual clinical conditions and subsequently underwent pacemaker implantation adhering to a standardized procedural protocol employing the technique previously delineated by Huang et al. (25), see Figure 2. The intracardiac electrogram (EGM) was connected to an electrophysiological multichannel recorder (Bard Electrophysiology Lab System, MA, USA), while a multi-lead surface ECG monitor was additionally attached before the procedure. The implantation details of the LBBaP procedure can be found in Supplementary Material S1. The criteria for assessing LBBaP included two distinct pacing modalities: left bundle branch pacing (LBBP) and left ventricular septal pacing (LVSP) or deep septal pacing. The diagnostic criteria for LBBaP in our study can also be found in Supplementary Material S1.

RVP: All procedures for RVP were conducted by skilled operators with extensive experience. An active-fixation pacing lead was meticulously positioned at the septum of the right ventricle. To validate the accurate placement of the right ventricle lead, fluoroscopic radiographs were obtained in a 45° left anterior oblique (LAO) view.

HBP: The procedural methods used in HBP are similar to those used in LBBaP, except that the target pacing site changes from the LBB to the His Bundle.

### Pacing parameter measurement

During the assessment, the unipolar configuration was employed to test the pacing threshold at a pulse width of 0.4 ms. Simultaneously, recordings were made of 12-lead surface ECG and intracardiac electrograms for all measurements. To determine the average R wave amplitude in the lead, measurements were repeated three times and the data were averaged. Final impedance value was established by averaging three separate measurements obtained during testing. Regular documentation of ventricular pacing (VP) burden was carried out for all patients. All three pacing modalities (LBBaP, HBP, and RVP) have employed the aforementioned measurement procedure.

### Evaluation of safety and prognosis

The safety evaluation encompassed adverse events that occurred during both the perioperative period and the follow-up. These included septal perforation, lead revision, pocket hematoma, pocket infection, and pericardial effusion.

Prognosis was evaluated based on heart failure hospitalization (HFH) and the primary composite outcome including death due to cardiovascular disease (CVD), HFH, or the necessity for upgrading to BVP during follow-up. After the implantation of pacemakers, subsequent in hospital follow-up clinic visits were organized at six-month intervals. During these visits, detailed medical records were meticulously gathered, and thorough physical assessments were performed by adept cardiologists. LVEF was assessed using the classical Teichholz method.

### Statistical analysis

Continuous variables were presented as the mean accompanied by the standard deviation (SD), alongside the median within the interquartile range if applicable. Paired comparisons were executed using the Student's *t*-test in cases where the data exhibited a normal or approximately normal distribution, while the Mann-Whitney *U*-test was employed for non-parametric data. Categorical data are presented as the number of cases and corresponding percentages. To examine differences in these categorical variables, statistical tests such as the *χ*^2^ test or Fisher exact test were employed. The Kaplan-Meier estimate was used to assess the occurrence of procedure-related complications, the primary composite outcome, and HFH, with the resulting *P*-values obtained from the Log-Rank test. All statistical tests were performed with a two-sided approach, and significance was determined by considering *P* values < 0.05. The management and analysis of the data were conducted employing the SPSS software, version 23.0, developed by IBM and originating from Chicago, Illinois.

## Results

### Baseline characteristics of LBBaP patients

Table 1 provides a comprehensive summary elucidating the baseline characteristics of 309 LBBaP patients included in the analysis. The cohort had an average age of 69.8 ± 8.7 years, with male patients constituting 57.0% of the total population. Among the patient population, 58.3% had a history of hypertension and 29.1% had documented AF. The mean baseline LVEF was 57.9% ± 11.6%, and the mean left ventricular end-diastolic dimension (LVEDD) was 49.1 ± 8.5 mm.

**Table 1:** Baseline characteristics of LBBaP patients between Non-CAD and stable CAD groups.

Variables	Overall (*N* = 309)	Non-CAD (*n* = 205)	Stable CAD (*n* = 104)	*P* value
Demographic features				
Age, years	69.8 ± 8.7	69.3 ± 9.1	70.9 ± 7.8	0.128
Male, *n* (%)	176 (57.0)	118 (57.6)	58 (55.8)	0.764
Indications, *n* (%)				
AVB, *n* (%)	146 (47.2)	96 (46.8)	50 (48.1)	0.836
SSS, *n* (%)	118 (38.2)	75 (36.6)	43 (41.3)	0.416
AF with long R-R interval, *n* (%)	47 (15.2)	35 (17.1)	12 (11.5)	0.200
Medications, *n* (%)				
Antiplatelet agents, *n* (%)	111 (35.9)	30 (14.6)	81 (77.9)	***<0***.001**
Oral anticoagulants, *n* (%)	73 (23.6)	50 (24.4)	23 (22.1)	0.656
Comorbidities				
Hypertension, *n* (%)	180 (58.3)	115 (56.1)	65 (62.5)	0.281
Diabetes, *n* (%)	72 (23.3)	40 (19.5)	32 (30.8)	***0***.027*
AF, *n* (%)	90 (29.1)	61 (29.8)	29 (27.9)	0.732
DCM, *n* (%)	14 (4.5)	8 (3.9)	6 (5.8)	0.317
HCM, *n* (%)	13 (4.2)	10 (4.9)	3 (2.9)	0.309
Laboratory data				
NT-proBNP, pg/ml	456.0 (136.0, 1017.0)	321.0 (120.0, 769.5)	724.5 (276.0, 1836.5)	***<0***.001**
eGFR, ml/min/1.73 m^2^	90.8 ± 22.0	92.0 ± 23.7	88.3 ± 18.1	0.162
Echocardiography				
LVEF, %	57.9 ± 11.6	58.2 ± 11.7	57.4 ± 11.3	0.564
LVEDD, mm	49.1 ± 8.5	48.9 ± 8.5	49.4 ± 8.5	0.621
IVS thickness, mm	10.9 ± 2.2	10.8 ± 2.2	11.1 ± 2.1	0.254

LBBaP, Left bundle branch area pacing; CAD, coronary artery disease; AVB, atrioventricular block; SSS, sick sinus syndrome; AF, atrial fibrillation; DCM, dilated cardiomyopathy; HCM, hypertrophic cardiomyopathy; NT-proBNP, N-terminal pro-brain natriuretic peptide; eGFR, estimated glomerular filtration fraction; LVEF, left ventricular ejection fraction; LVEDD, left ventricular end-diastolic dimension; IVS, interventricular septum.

Bold and italics values indicate that *P* < 0.05.

* *P* < 0.05.

** *P* < 0.001.

The leading reasons for pacemaker implantation were AVB (47.2%) and SSS (38.2%). Within the patient population, those with stable CAD presented a higher utilization of antiplatelet agents (77.9% vs. 14.6%, *P* < 0.001) and a greater prevalence of diabetes (30.8% vs. 19.5%, *P* = 0.027) in comparison to individuals with non-CAD. Furthermore, although the comparable LVEF between the two groups (*P* = 0.564), patients with stable CAD had significantly elevated concentrations of N-terminal pro-brain natriuretic peptide (NT-proBNP) in contrast to individuals without CAD (*P* < 0.001), with mean values and 95% confidence intervals (CI) of 724.5 [276.0, 1836.5] pg/ml and 321.0 [120.0, 769.5] pg/ml, respectively. For stable CAD patients, the proportion of single vessel disease was 78.8%, with stent implantation observed in 51.0% of cases. The mean value and 95% CI of cardiac troponin I (cTnI) were measured at 0.011 (0.005, 0.032) ng/ml. Additional information regarding CAG/PCI procedure data for stable CAD patients can be found in Supplementary Table S1.

### Periprocedural measurements and exploratory analysis of LBBaP patients

Table 2 shows the procedural details and complications in the study participants. The total procedural time and duration of fluoroscopy were similar in both groups. The LBB potential, stimulus to left ventricular activation time (Sti-LVAT) and paced QRSd were also comparable between the two groups. After lead fixation, the R-wave amplitude showed no significant difference between the two cohorts (11.6 ± 3.7 mV vs. 12.3 ± 5.0 mV, *P* = 0.181). Stable CAD patients, in contrast to patients without CAD, had higher pacing thresholds (0.71 ± 0.30 V vs. 0.66 ± 0.26 V, *P* = 0.127) and impedances (772.8 ± 216.1Ω vs. 739.2 ± 193.6*Ω*, *P* = 0.167). However, these differences did not reach statistical significance. Furthermore, no statistically significant disparities were detected between the two groups in terms of pacing thresholds, R wave amplitudes, and impedances at 6 months and 12 months follow-up. Regarding complications, a total of 16 patients (5.2%) experienced procedure-related complications in this cohort, with a substantially greater incidence of pocket hematoma when comparing the stable CAD group to the non-CAD group (4.8% vs. 0.5%, *P* = 0.030). This was likely associated with administration of antiplatelet agents.

**Table 2:** LBBaP characteristics during the procedure and follow-up between Non-CAD and stable CAD groups.

Parameters	Overall (*N* = 309)	Non-CAD (*n* = 205)	Stable CAD (*n* = 104)	*P* value
Total procedural time, min	99.2 ± 39.4	97.8 ± 41.3	101.9 ± 35.3	0.395
Total Fluoroscopy Time, min	10.3 (8.0, 13.3)	10.2 (8.2, 12.6)	10.6 (7.3, 13.7)	0.638
Preprocedural measurements				
Preimplant QRS duration, ms	108.7 ± 30.5	107.6 ± 29.5	110.8 ± 32.4	0.391
LBBB, *n* (%)	25 (8.1)	13 (6.3)	12 (11.5)	0.113
RBBB, *n* (%)	38 (12.3)	28 (13.7)	10 (9.6)	0.306
Intraprocedural measurements				
Sti-LVAT, ms	72.8 ± 10.1	72.4 ± 10.1	73.5 ± 9.9	0.340
Paced QRS duration, ms	115.1 ± 11.8	114.2 ± 11.0	116.7 ± 13.1	0.080
LBB potential, *n* (%)	207 (67.0)	131 (63.9)	76 (73.1)	0.105
Pacing threshold, V/0.4 ms	0.67 ± 0.27	0.66 ± 0.26	0.71 ± 0.30	0.127
R wave amplitude, mV	12.1 ± 4.6	12.3 ± 5.0	11.6 ± 3.7	0.181
Impedance, Ω	750.5 ± 201.7	739.2 ± 193.6	772.8 ± 216.1	0.167
6-month follow-up				
Pacing threshold, V/0.4 ms	0.70 ± 0.29	0.68 ± 0.29	0.73 ± 0.28	0.128
R wave amplitude, mV	13.8 ± 5.0	14.1 ± 5.3	13.2 ± 4.4	0.111
Impedance, Ω	617.2 ± 174.8	604.4 ± 183.7	642.4 ± 153.4	0.071
12-month follow-up	*N* = 241	*N* = 158	*N* = 83	
Pacing threshold, V/0.4 ms	0.72 ± 0.30	0.71 ± 0.31	0.75 ± 0.28	0.254
R wave amplitude, mV	13.8 ± 5.0	13.9 ± 5.1	13.5 ± 4.7	0.459
Impedance, Ω	627.2 ± 164.4	622.1 ± 175.9	637.3 ± 139.1	0.443
Follow-up	*N* = 309	*N* = 205	*N* = 104	
Primary composite outcome	15 (4.9)	8 (3.9)	7 (6.7)	0.274
HFH, *n* (%)	12 (3.9)	7 (3.4)	5 (4.8)	0.376
Upgrade to BVP, *n* (%)	0 (0.0)	0 (0.0)	0 (0.0)	1.000
Death due to CVD, *n* (%)	3 (1.0)	1 (0.5)	2 (1.9)	0.547
Procedure-related complications	16 (5.2)	8 (3.9)	8 (7.7)	0.155
Septal perforation, *n* (%)	2 (0.6)	2 (1.0)	0 (0.0)	0.552
Lead revision, *n* (%)	3 (1.0)	2 (1.0)	1 (1.0)	0.990
Pocket hematoma, *n* (%)	6 (1.9)	1 (0.5)	5 (4.8)	***0*** **.** ***030*** *
Pocket infection, *n* (%)	2 (0.6)	1 (0.5)	1 (1.0)	0.624
Pericardial effusion, *n* (%)	3 (1.0)	2 (1.0)	1 (1.0)	0.990

LBBaP, left bundle branch area pacing; CAD, coronary artery disease; LBBB, left bundle branch block; RBBB, right bundle branch block; Sti-LVAT, stimulus to left ventricular activation time; LBB, left bundle branch; HFH, heart failure hospitalization; BVP, biventricular pacing; CVD, cardiovascular disease.

Bold and italics values indicate that *P* < 0.05.

* *P* < 0.05.

After stratifying based on the number of diseased vessels and the vascular locations of single vessel disease in stable CAD patients, an assessment was made to determine the differences in pacing parameters. Exploratory analysis revealed that although pacing parameters for LAD/LM group were slightly worse to LCX and RCA groups, no statistically significant differences were observed at baseline, 6 months, and 1 year (Supplementary Table S2). Furthermore, no apparent differences between patients with single vessel disease and those with multivessel disease were observed at baseline, 6 months, and 1 year (Supplementary Table S3).

### Evaluation of safety and prognosis of LBBaP patients

The follow-up results revealed that non-CAD cohort with concurrent LBBB exhibited a statistically significant improvement in LVEF (*P* < 0.001) and a notable reduction in LVEDD (*P* < 0.001) during the 12-month follow-up period compared to baseline, while non-CAD cohort without LBBB did not show statistically significant differences (*P* = 0.100; *P* = 0.082) (Supplementary Table S4). Additionally, similar results were also observed in stable CAD patients (Supplementary Table S5).

**Figure 3:**
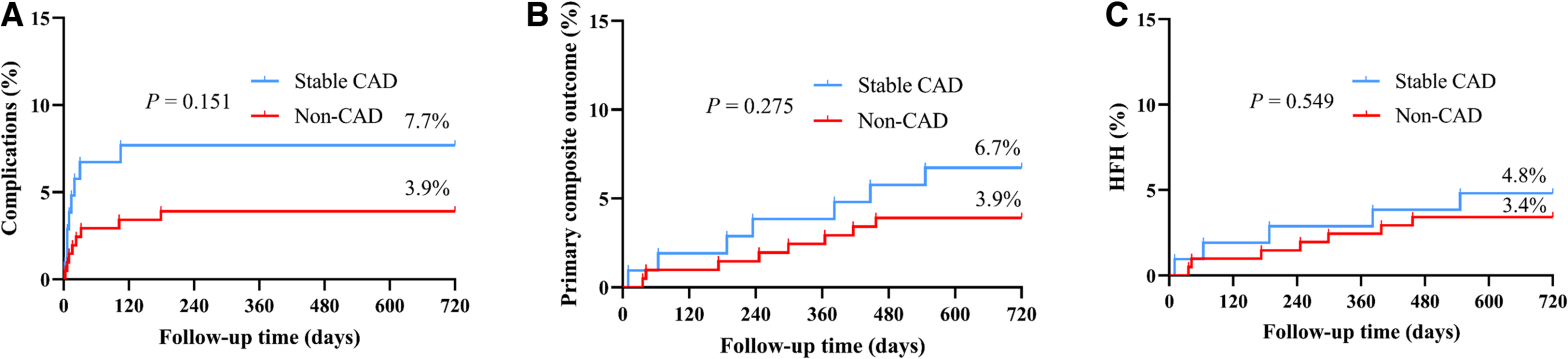
The Kaplan–Meier survival curves and analysis of procedure-related complications (**A**), primary composite outcome (**B**), and HFH (**C**) between stable CAD and Non-CAD groups who underwent LBBaP implantation. HFH, heart failure hospitalization; CAD, coronary artery disease; LBBaP, left bundle branch area pacing; *P < 0.05.

Table 2 and Figure 3 provides additional information on the safety and prognosis pertaining to the patients under investigation in the study. According to the data presented in Table 2, after a follow-up of 17.4 ± 5.3 months, the overall incidence of procedure-related complications was 3.9% in the non-CAD cohort and 7.7% in the stable CAD cohort (*P* = 0.155). The primary composite outcome, including death due to CVD, HFH, or the necessity for upgrading to BVP, was not significantly different between the two groups with a rate of 3.9% in non-CAD group and 6.7% in stable CAD group. The incidence of HFH and death due to CVD was slightly higher in the stable CAD group but, similarly, the observed differences did not reach statistical significance (4.8% vs. 3.4%, *P* = 0.376).

Figure 3 shows the Kaplan–Meier analysis of procedure-related complications, the primary composite outcome, and HFH. The results indicate comparable probabilities for experiencing procedure-related complications or the primary composite outcome between two groups. While the probability of experiencing HFH was slightly higher in the stable CAD group, the difference failed to reach statistical significance.

### Baseline and follow-up among LBBaP, HBP, and RVP groups combined with stable CAD

Three hundred twenty-one patients with stable CAD were enrolled in the study. Among them, LBBaP was performed in 104 patients, and HBP was conducted in 64 patients, while 153 patients underwent RVP. In accordance with the data presented in Supplementary Table S6, patients among the three groups exhibited comparable average age, the proportion of men, preimplant QRSd, NT-proBNP, diabetes, AF, AVB, and other comorbidities except for the prevalence of hypertension (RVP vs. LBBaP, 49.0% vs. 62.5%, *P* = 0.033; RVP vs. HBP, 49.0% vs. 65.6%, *P* = 0.025). Baseline LVEF was also comparable among the LBBaP group (57.4% ± 11.3%), HBP group (60.2% ± 10.0%), and RVP group (58.6% ± 11.3%). In terms of the severity of CAD, there were also no apparent differences among the three groups in the incidence of multivessel disease and levels of cTnI. Overall, all groups demonstrated a reasonable level of comparability, with the RVP group exhibiting a lower prevalence of hypertension.

**Figure 4:**
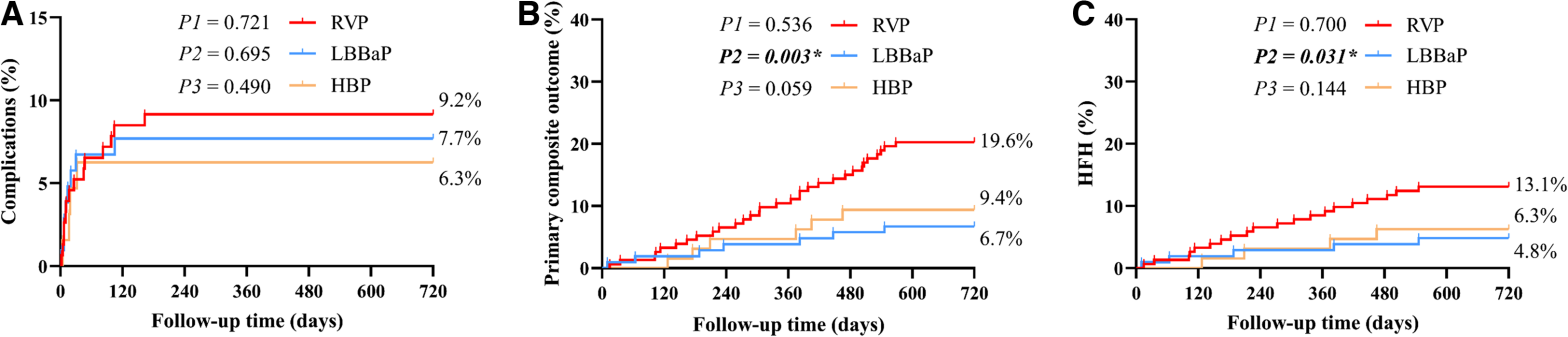
The Kaplan–Meier survival curves and analysis of procedure-related complications (**A**), primary composite outcome (**B**), and HFH (**C**) among the three groups combined with stable CAD. RVP, right ventricular pacing; LBBaP, left bundle branch area pacing; HBP, his-bundle pacing; HFH, heart failure hospitalization; CAD, coronary artery disease; P1: LBBaP vs. HBP; P2: LBBaP vs. RVP; P3: HBP vs. RVP; *P < 0.05.

Table 3 displays the pacing parameters assessed throughout both the procedural phase and subsequent follow-up period, which demonstrate that the HBP group had relatively lower R wave amplitudes and higher pacing thresholds compared to the RVP group and the LBBaP group at baseline, 6-month and 12-month follow-up assessments. In addition, the LBBaP group had better R wave amplitudes than the RVP group at baseline and 6-month follow-up. Of particular note, the RVP group exhibited a markedly higher paced QRSd compared to both the LBBaP and HBP groups (Table 3). Figure 4 illustrates the Kaplan–Meier analysis of procedure-related complications, the primary composite outcome, and HFH in the three groups. As noted above, the incidence of procedure-related complications failed to reach statistical significance among the LBBaP, HBP, and RVP groups. Nevertheless, the RVP group had significantly higher rates of the primary composite outcome (19.6% vs. 6.7%, *P* = 0.003) and HFH (13.1% vs. 4.8%, *P* = 0.031) than LBBaP group (Figure 4). The RVP group also demonstrated significantly higher rates of upgrade to BVP events than LBBaP group (3.9% vs. 0.0%, *P* = 0.041) (Table 4). Notably, there were no significant disparities observed between the HBP group and the LBBaP group in terms of the incidence of procedure-related complications, the primary composite outcome, and HFH. Furthermore, within the stable CAD population undergoing LBBaP, there were 70 (67.3%) individuals who underwent LBBP, while 34 (32.7%) individuals received LVSP. No statistically significant differences were observed in terms of safety and prognosis between these two pacing modalities in stable CAD patients (Supplementary Table S7).

**Table 3:** Pacing parameters among LBBaP, HBP, and RVP groups combined with stable CAD.

Parameters	LBBaP (*N* = 104)	HBP (*N* = 64)	RVP (*N* = 153)	*P1*	*P2*	*P3*
Paced QRS duration	116.7 ± 13.1	114.5 ± 13.7	152.7 ± 36.1	0.597	***<0*** **.** ***001*** **	***<0*** **.** ***001*** **
Baseline						
Pacing threshold, V/0.4 ms	0.71 ± 0.30	1.18 ± 0.43	0.78 ± 0.29	***<0*** **.** ***001*** **	0.089	***<0*** **.** ***001*** **
R wave amplitude, mV	11.6 ± 3.7	5.7 ± 3.2	9.4 ± 3.7	***<0*** **.** ***001*** **	***<0*** **.** ***001*** **	***<0*** **.** ***001*** **
Impedance, Ω	772.8 ± 216.1	835.6 ± 238.7	812.4 ± 258.1	0.102	0.198	0.519
6-month follow-up						
Pacing threshold, V/0.4 ms	0.73 ± 0.28	1.23 ± 0.39	0.79 ± 0.28	***<0*** **.** ***001*** **	0.119	***<0*** **.** ***001*** **
R wave amplitude, mV	13.2 ± 4.4	6.4 ± 2.2	10.2 ± 3.2	***<0*** **.** ***001*** **	***<0*** **.** ***001*** **	***<0*** **.** ***001*** **
Impedance, Ω	642.4 ± 153.4	693.5 ± 208.3	677.6 ± 176.8	0.069	0.117	0.546
12-month follow-up	*N* = 83	*N* = 50	*N* = 127		* *	* *
Pacing threshold, V/0.4 ms	0.75 ± 0.28	1.25 ± 0.37	0.81 ± 0.28	***<0*** **.** ***001*** **	0.145	***<0*** **.** ***001*** **
R wave amplitude, mV	13.5 ± 4.7	8.2 ± 2.5	12.6 ± 4.4	***<0*** **.** ***001*** **	0.112	***<0*** **.** ***001*** **
Impedance, Ω	637.3 ± 139.1	672.4 ± 185.3	658.2 ± 148.4	0.151	0.285	0.533

LBBaP, left bundle branch area pacing; HBP, his-bundle pacing; RVP, right ventricular pacing; CAD, coronary artery disease; P1: LBBaP vs. HBP; P2: LBBaP vs. RVP; P3: HBP vs. RVP.

Bold and italics values indicate that *P* < 0.05.

** *P* < 0.001.

**Table 4:** Safety and prognosis among LBBaP, HBP, and RVP groups combined with stable CAD.

Events	LBBaP	HBP	RVP	*P1*	*P2*	*P3*
All patients	*N* = 104	*N* = 64	*N* = 153			
Primary composite outcome	7 (6.7)	6 (9.4)	30 (19.6)	0.535	***0*** **.** ***004*** *	0.065
HFH, *n* (%)	5 (4.8)	4 (6.3)	20 (13.1)	0.688	***0*** **.** ***029*** *	0.145
Upgrade to BVP, *n* (%)	0 (0.0)	1 (1.6)	6 (3.9)	0.202	***0*** **.** ***041*** *	0.371
Death due to CVD, *n* (%)	2 (1.9)	1 (1.6)	4 (2.6)	0.864	0.719	0.638
VP burden > 20%	*N* = 79	*N* = 46	*N* = 95			
Primary composite outcome	6 (7.6)	4 (8.7)	25 (26.3)	0.828	***0*** **.** ***001*** *	***0*** **.** ***016*** *
HFH, *n* (%)	4 (5.1)	2 (4.3)	16 (16.8)	0.857	***0*** **.** ***016*** *	***0*** **.** ***038*** *
Upgrade to BVP, *n* (%)	0 (0.0)	1 (2.2)	6 (6.3)	0.190	***0*** **.** ***023*** *	0.290
Death due to CVD, *n* (%)	2 (2.5)	1 (2.2)	3 (3.2)	0.900	0.806	0.742
VP burden ≤ 20%	*N* = 25	*N* = 18	*N* = 58			
Primary composite outcome	1 (4.0)	2 (11.1)	5 (8.6)	0.372	0.459	0.751
HFH, *n* (%)	1 (4.0)	2 (11.1)	4 (6.9)	0.372	0.613	0.565
Upgrade to BVP, *n* (%)	0 (0.0)	0 (0.0)	0 (0.0)	1.000	1.000	1.000
Death due to CVD, *n* (%)	0 (0.0)	0 (0.0)/	1 (1.7)	1.000	0.511	0.577
VP burden > 40%	*N* = 68	*N* = 42	*N* = 81			
Primary composite outcome	5 (7.4)	4 (9.5)	21 (25.9)	0.688	***0*** **.** ***003*** *	***0*** **.** ***033*** *
HFH, *n* (%)	3 (4.4)	2 (4.8)	13 (16.0)	0.932	***0*** **.** ***023*** *	0.071
Upgrade to BVP, *n* (%)	0 (0.0)	1 (2.4)	5 (6.2)	0.203	***0*** **.** ***038*** *	0.357
Death due to CVD, *n* (%)	2 (2.9)	1 (2.4)	3 (3.7)	0.862	0.798	0.696
VP burden ≤ 40%	*N* = 36	*N* = 22	*N* = 72			
Primary composite outcome	2 (5.6)	2 (9.1)	9 (12.5)	0.609	0.263	0.665
HFH, *n* (%)	2 (5.6)	2 (9.1)	7 (9.7)	0.609	0.462	0.930
Upgrade to BVP, *n* (%)	0 (0.0)	0 (0.0)	1 (1.4)	1.000	0.480	0.580
Death due to CVD, *n* (%)	0 (0.0)	0 (0.0)/	1 (1.4)	1.000	0.480	0.580
	*N* = 104	*N* = 64	*N* = 153			
Procedure-related complications	8 (7.7)	4 (6.3)	14 (9.2)	0.725	0.682	0.481
Septal perforation, *n* (%)	0 (0.0)	0 (0.0)	1 (0.7)	1.000	0.410	0.518
Lead revision, *n* (%)	1 (1.0)	0 (0.0)	1 (0.7)	0.433	0.783	0.518
Pocket hematoma, *n* (%)	5 (4.8)	3 (4.7)	9 (5.9)	0.972	0.710	0.726
Pocket infection, *n* (%)	1 (1.0)	1 (1.6)	2 (1.3)	0.728	0.800	0.883
Pericardial effusion, *n* (%)	1 (1.0)	0 (0.0)	1 (0.7)	0.433	0.783	0.518

LBBaP, left bundle branch area pacing; HBP, his-bundle pacing; RVP, right ventricular pacing; CAD, coronary artery disease; HFH, heart failure hospitalization; BVP, biventricular pacing; CVD, cardiovascular disease; VP, ventricular pacing; P1: LBBaP vs. HBP; P2: LBBaP vs. RVP; P3: HBP vs. RVP.

Bold and italics values indicate that *P* < 0.05.

* *P* < 0.05.

**Figure 5:**
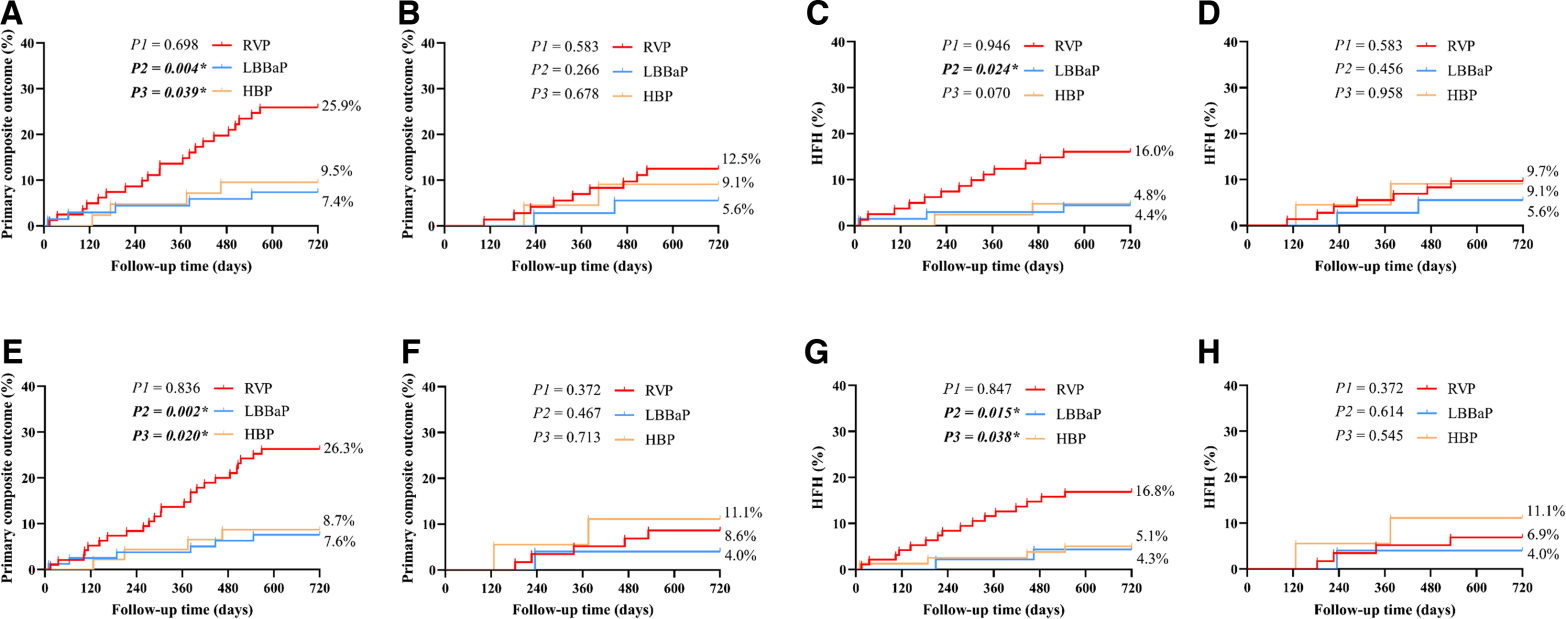
The subgroup Kaplan–Meier survival curves and analysis of primary composite outcome and HFH among the three groups combined with stable CAD. The primary composite outcome and HFH were analyzed according to VP burden > 40% (**A,C**), VP burden ≤ 40% (**B,D**), VP burden > 20% (**E,G**), and VP burden ≤ 20% (**F,H**). RVP, right ventricular pacing; LBBaP, left bundle branch area pacing; HBP, his-bundle pacing; HFH, heart failure hospitalization; CAD, coronary artery disease; VP, ventricular pacing; P1: LBBaP vs. HBP; P2: LBBaP vs. RVP; P3: HBP vs. RVP; *P < 0.05.

Stable CAD patients were further categorized and subjected to analysis according to their VP burden as documented at the conclusion of the follow-up period (Figure 5). Within the cohort of patients exhibiting a VP burden exceeding 40%, the RVP group had significantly higher rates of the primary composite outcome (25.9% vs. 7.4%, *P* = 0.004) and HFH (16.0% vs. 4.4%, *P* = 0.024) compared to the LBBaP group. However, no substantial differences were detected between these two groups for patients with a VP burden ≤ 40% (primary composite outcome, *P* = 0.266; HFH, *P* = 0.456). When stratified by VP > 20%, the RVP group also exhibited a similarly higher incidence of the primary composite outcome (26.3% vs. 7.6%, *P* = 0.002) and HFH (16.8% vs. 4.3%, *P* = 0.015) compared to the LBBaP group. Conversely, when VP ≤ 20%, no significant statistical differences were observed between the two groups (primary composite outcome, *P* = 0.467; HFH, *P* = 0.614). Additionally, the HBP group had significantly lower rates of the primary composite outcome compared to the RVP group among patients with VP burden exceeding both 40% (9.5% vs. 25.9%, *P* = 0.039) and 20% (8.7% vs. 26.3%, *P* = 0.020).

## Discussion

Our retrospective study included patients with symptomatic bradycardia who underwent LBBaP and divided them into 104 patients with stable CAD and 205 patients with non-CAD according to CAG. Our findings demonstrated that LBBaP, as a representative of physiological pacing, can be safely implemented in stable CAD patients without a significant increase in the risk of procedure-related complications and that mid-term prognosis of stable CAD patients who underwent LBBaP was similar to non-CAD patients. Additionally, to further verify the effectiveness of LBBaP in stable CAD patients, our study also included 64 stable CAD patients who underwent HBP and 153 stable CAD patients who underwent RVP to compare with the 104 stable CAD patients who underwent LBBaP. Our findings also demonstrated that for stable CAD patients, permanent LBBaP seems to result in more stable pacing parameters, similar procedural complications, and a midterm prognosis comparable to HBP. In comparison with RVP, LBBaP might have similar pacing parameters, comparable procedural complications, and a better midterm prognosis. In conclusion, we believe permanent LBBaP is feasible and effective for stable CAD patients.

Stable CAD is among the major global disease burdens, especially in the context of an aging population (26). Stable CAD patients should be recognized as a distinct population cohort, given their worse quality of life and a propensity for adverse outcomes (27, 28). Especially when patients receive pacemaker implantation, CAD patients have been reported to experience myocardial ischemia and an elevated susceptibility to bleeding associated with the administration of antiplatelet agents, resulting in an increased risk of complications such as unstable pacing parameters, pocket hematoma, and pocket infection (22, 29–31). Therefore, our investigation into the safety and prognostic implications of pacemaker implantation in stable CAD patients has significant clinical importance.

Throughout our investigation, the presence of an LBB potential was recorded in 67.0% of the individuals examined, with 63.9% displaying potential in the non-CAD group and 73.1% in the stable CAD group. This is consistent with the 50% to 80% range of LBB potential recordings reported in previous studies (32–34). Additionally, our research found that stable CAD patients had similar pacing thresholds, R wave amplitudes, and impedances compared to non-CAD patients during the procedure and at the 6-month and 12-month follow-up visits. Previous studies have presented divergent accounts concerning the impact of myocardial ischemia on pacing parameters (20, 35–37). Considering that, our study population included patients who underwent pacemaker implantation and CAG during the same hospitalization period. These patients did not have particularly severe clinical manifestations of acute coronary syndrome ACS at admission, but rather exhibited a stable chronic myocardial ischemic state. It is plausible that CAD without acute myocardial ischemia or myocardial scars caused by myocardial infarction may not affect pacing parameters. This observation provides a potential explanation for the findings of our exploratory analysis. Despite the essential role of LAD/LM in supplying the interventricular septum, no significant disparities in pacing parameters were observed among the LAD/LM, LCX, and RCA groups in the single vessel disease population.

During our safety evaluation, we analyzed a follow up period that spanned 17.4 ± 5.3 months. The overall prevalence of procedure-related complications observed in both groups was low, especially the incidence of lead-related complications, which aligns with previous investigations (33, 38). Jastrzębski et al. documented a total of 38 instances of LBBaP lead dislodgement in 2,533 patients (39) and Wang et al. reported 2 instances of LBBaP lead revision in 406 patients (40). Their findings demonstrated an incidence rate similar to what was found in our study. Previous investigations have demonstrated that antiplatelet agents, particularly dual antiplatelet agents, are an independent risk factor for the pocket hematoma (21, 22). In our study, the proportion of stable CAD patients using antiplatelet agents was significantly higher than non-CAD patients, which likely accounts for the increased incidence of pocket hematoma observed in stable CAD cohort relative to non-CAD cohort. Overall, the safety of LBBaP implantation in stable CAD patients appears to be acceptable when performed in experienced centers, but these findings also require additional confirmation from a study with a larger sample size. LBBaP has the potential to achieve physiological conduction, mechanical synchrony, and correction of LBBB, which could contribute to improved clinical outcomes in patients with bradycardia (16, 41, 42). Consistent with prior research findings, our study also showed that LBBaP may result in similar cardiac functional improvement for both stable CAD and non-CAD patients with concomitant LBBB (43, 44).

Previous studies have shown that HBP may exhibit relatively lower R wave amplitudes and higher pacing thresholds compared to RVP and LBBaP (13, 45, 46), which is consistent with our findings. This finding also suggests that LBBaP may have more stable pacing parameters compared to HBP, which may also be applicable to the stable CAD population. Sharma et al. reported that LBBaP might have lower mortality, HFH, and their primary composite outcome (all-cause mortality, HFH, or upgrade to BVP) compared to RVP (47). In our study, LBBaP was associated with improved HFH and primary composite outcome, but it did not show a significant improvement in mortality rate due to CVD, which may be attributed to their extended duration of follow-up (mean follow-up 19.4 ± 9.0 months) compared to 17.4 ± 5.3 months in our study. Additionally, previous research has demonstrated that VP burden is an independent risk factor for unfavorable cardiovascular outcomes in individuals with pacemakers (47–49). Consequently, we stratified our study cohort based on a VP burden > 40%, and the results showed that in the subgroup with VP burden > 40%, both the incidence of HFH and primary composite outcome were significantly higher in RVP group than in LBBaP group, while no statistically significant difference was noted in the VP burden ≤ 40% subgroup. When stratifying our population based on a threshold of 20% for VP similar results were also obtained. Notably, these findings are consistent with earlier studies conducted in this field (3, 47, 50). The primary composite outcome discrepancy between the RVP group and the LBBaP group was principally driven by HFH, especially in patients with higher burdens of VP. The plausible mechanism underlying the decrease in these adverse outcomes could be attributed to the preservation of synchronous ventricular activation through LBBaP, which prevented dyssynchrony-mediated adverse consequences observed in the RVP cohort (15, 51). Although in this study, the HBP group also achieved the similar incidence of HFH and primary composite outcome as the LBBaP group in the stable CAD population, considering the instability of HBP parameters, LBBaP may be a more suitable pacing modality for stable CAD patients with bradycardia, particularly those with higher burdens of VP.

### Study limitations

This study has several limitations, primarily stemming from its retrospective and observational design. As such, the non-randomized design limits the ability to establish causality or control for potential confounding factors, and there may be unmeasured variables that were not accounted for in the analysis. Although most variables exhibited no notable differences between groups at baseline, the lack of randomization introduces the potential for selection bias and confounding. Hence, prudence is advised in interpreting the findings of this study. Furthermore, our study merely affirms the feasibility and safety of LBBaP in stable CAD. While this population represents the main CAD group undergoing pacemaker implantation and CAG during the same hospitalization in clinical practice, it is important to acknowledge that the potential alterations in pacing parameters and clinical prognosis of LBBaP when confronted with acute coronary syndrome, particularly in cases of severe stenosis in the LAD/LM, are currently unknown. Investigating whether any changes occur in LBBaP parameters following the utilization of PCI to alleviate stenosis is of utmost importance. This information would further assist clinicians in making prudent decisions regarding the management of patients with concomitant CAD undergoing LBBaP. Although patients with such conditions are relatively rare, preliminary data may potentially be obtained in advance through animal models.

## Conclusion

Compared with non-CAD patients, LBBaP was found to be attainable in stable CAD patients and exhibited comparable mid-term safety and prognosis. Furthermore, in the stable CAD population, LBBaP has demonstrated more stable pacing parameters than HBP, and better prognostic outcomes compared to RVP.

## Data Availability

The original contributions presented in the study are included in the article/**Supplementary Materials**, further inquiries can be directed to the corresponding author/s.
