# Supplementary material for: Feasibility and safety of left bundle branch area pacing for patients with stable coronary artery disease
Source: Front Cardiovasc Med. 2023 Nov 30;10:1246846. doi: 10.3389/fcvm.2023.1246846 (PMC10720039; doi:10.3389/fcvm.2023.1246846)
Supplement: Supplementary file 1 [file Datasheet1.docx]

Feasibility and Safety of Left Bundle Branch Area Pacing for Patients with Stable Coronary Artery Disease

Yu Shan, MD^1,2,†^, Maoning Lin, MD^1,2,†^, Xia Sheng, MD, PHD^1,2^, Jiefang Zhang, MD, PHD^1,2^, Yaxun Sun, MD, PHD ^1,2^, Guosheng Fu, MD, PHD ^1,2,*^, Min Wang, MD, PHD ^1,2,*^

^1^Department of Cardiology, Sir Run Run Shaw Hospital, College of Medicine, Zhejiang University, No 3 East of Qinchun Road, Hangzhou, Zhejiang 310000, China

^2^Key Laboratory of Cardiovascular Intervention and Regenerative Medicine of Zhejiang Province, Hangzhou, China

† Yu Shan and Maoning Lin have contributed equally to this work and share the first authorship.

* Min Wang and Guosheng Fu are the corresponding authors of this article.

*** Correspondence:**Min Wang, MD PhD
wangminsyf30508@zju.edu.cn

Keywords: Left bundle branch area pacing (LBBaP); Stable coronary artery disease; Pacing parameters; Heart failure hospitalization (HFH); Safety.

**Supplementary material**

**Supplementary Material 1 The detailed procedure for LBBaP and LBBaP criteria.**

**P3**

**Supplementary Table 1 The CAG/PCI procedure data of stable CAD patients who underwent LBBaP. P4**

**Supplementary Table 2 Exploratory analysis of pacing parameters based on different vascular locations in patients with single vessel disease who underwent LBBaP. P5**

**Supplementary Table 3 Exploratory analysis of pacing parameters based on the number of diseased vessels in patients with stable CAD who underwent LBBaP. P6**

**Supplementary Table 4 Echocardiographic evaluation of patients with Non-CAD following LBBaP implantation, stratified by the presence or absence of LBBB. P7**

**Supplementary Table 5 Echocardiographic evaluation of patients with stable CAD following LBBaP implantation, stratified by the presence or absence of LBBB. P8**

**Supplementary Table 6 Baseline characteristics among LBBaP, HBP, and RVP groups combined with stable CAD. P9**

**Supplementary Table 7 Evaluation of the safety and prognosis in stable CAD patients under the pacing modalities of LBBP and LVSP. P10**

**Supplementary Material 1 The detailed procedure for LBBaP and LBBaP criteria.**

**The detailed procedure for LBBaP**

In a concise summary, a C315 sheath (manufactured by Medtronic, Inc., Minneapolis, MN) was employed to ascertain the initial fixation site situated at a right anterior oblique (RAO) position of 20-30°. Mapping of the His bundle region was accomplished by employing the pacing lead in a unipolar configuration, which served as a fluoroscopic reference point. Then, a gradual advancement of both the pacing lead and sheath by 1-2 cm towards the apex was carried out in the RAO view, accompanied by a slight counterclockwise rotation. This maneuver aimed to guide the pacing lead towards the septum, where a distinctive W-shaped pattern was observed in the V1 lead. Notably, when pacing at a 2 V output using a unipolar configuration from the distal electrode of the 3830 lead, the notch in the waveform was positioned proximate to the nadir. Subsequently, a controlled counterclockwise rotation of the C315 sheath was typically performed, ensuring that the sheath tip was oriented perpendicularly to the interventricular septum. Additionally, employing a fluoroscopy left anterior oblique (LAO) position ranging from 30 to 45 degrees, the electrode was meticulously screwed into the interventricular septum, as depicted in Figure 2. Employing a systematic clockwise rotation, the lead body underwent gradual and incremental advancement of the lead tip through the interventricular septum, while intermittently pacing the tip electrode to carefully observe modifications in the V1 QRS morphology. Subsequently, the previously observed W-shaped "notch" underwent a gradual displacement until the delayed manifestation of the R wave within the QRS complex materialized, indicative of a consistent correlation with the presence of a right bundle branch block (RBBB).

**LBBaP criteria**

Basically, LBBaP criteria are may include 2 different pacing modalities: Left bundle branch pacing (LBBP): left bundle branch (LBB) capture can be determined by the presence of a RBBB pattern paced morphology and one or more of the following criteria: 1) visualization of left bundle potential and evidence of left bundle capture; 2) transition from non-selective to selective left bundle capture or non-selective to left ventricular septal myocardial capture during threshold testing; 3) Abrupt shortening of stimulus to left ventricular activation time (Sti-LVAT) > 10ms, measured from the pacing spike to the peak of lead V5 or V6. Another situation which was also belong to LBBaP was left ventricular septal pacing (LVSP) or deep septal pacing: the pacing lead is placed deep in the left septal subendocardium but no LBB capture criteria can be demonstrated. A RBBB pattern paced QRS morphology is also usually present. Typically, Sti-LVAT in V6 will be > 80ms but usually < 90ms, and no changes in paced QRS morphology will be seen during pacing threshold evaluation.

**Supplementary Table 1 The CAG/PCI procedure data of stable CAD patients who underwent LBBaP.**

| **CAG/PCI procedure data** | **CAD (N=104)** |
| --- | --- |
| Lesion location, n (%) |  |
| LM, n (%) | 5 (4.8) |
| LAD, n (%) | 65 (62.5) |
| LCX, n (%) | 29 (27.9) |
| RCA, n (%) | 33 (31.7) |
| Single vessel disease, n (%) | 82 (78.8) |
| Multivessel disease, n (%) | 22 (21.2) |
| Stent implantation, n (%) | 53 (51.0) |
| Total length of stents, mm | 31.0 (18.0, 47.0) |
| cTnI, ng/ml | 0.011 (0.005, 0.032) |

CAG: coronary angiography; PCI: percutaneous coronary intervention; CAD: coronary artery disease; LBBaP: left bundle branch area pacing; LM: left main coronary artery; LAD: left anterior descending artery; LCX: left circumflex artery; RCA: right coronary artery; cTnI: cardiac troponin I.

**Supplementary Table 2 Exploratory analysis of pacing parameters based on different vascular locations in patients with single vessel disease who underwent LBBaP.**

|  | Single vessel disease | | |  | | |
| --- | --- | --- | --- | --- | --- | --- |
|  | LAD/LM (n=45) | LCX (n=17) | RCA (n=20) | *P1* | *P2* | *P3* |
| **Intraprocedural measurements** |  |  |  |  |  |  |
| Pacing threshold, V/0.4 ms | 0.72 ± 0.24 | 0.67 ± 0.27 | 0.66 ± 0.24 | 0.491 | 0.946 | 0.415 |
| R-wave amplitude, mV | 11.4 ± 3.4 | 12.3 ± 3.9 | 12.7 ± 4.4 | 0.403 | 0.757 | 0.205 |
| Impedance, Ω | 777.3 ± 191.4 | 745.7 ± 218.8 | 723.3 ± 210.0 | 0.593 | 0.744 | 0.332 |
| **6-month follow-up** |  |  |  |  |  |  |
| Pacing threshold, V/0.4 ms | 0.74 ± 0.23 | 0.68 ± 0.26 | 0.70 ± 0.26 | 0.400 | 0.819 | 0.539 |
| R-wave amplitude, mV | 13.0 ± 4.4 | 14.1 ± 4.6 | 13.6 ± 4.4 | 0.386 | 0.694 | 0.662 |
| Impedance, Ω | 640.0 ± 125.8 | 619.5 ± 144.3 | 613.1 ± 132.1 | 0.593 | 0.885 | 0.456 |
| **12-month follow-up** | **N = 37** | **N = 12** | **N = 14** |  |  |  |
| Pacing threshold, V/0.4 ms | 0.75 ± 0.23 | 0.70 ± 0.28 | 0.71 ± 0.28 | 0.507 | 0.897 | 0.585 |
| R-wave amplitude, mV | 12.8 ± 4.1 | 14.3 ± 5.3 | 13.9 ± 5.5 | 0.277 | 0.761 | 0.433 |
| Impedance, Ω | 637.4 ± 138.9 | 606.3 ± 74.4 | 622.9 ± 102.3 | 0.379 | 0.686 | 0.661 |

LBBaP: left bundle branch area pacing. LAD: left anterior descending artery; LM: left main coronary artery; LCX: left circumflex artery; RCA: right coronary artery; P1: LAD vs LCX; P2: LCX vs RCA; P3: LAD vs RCA.

**Supplementary Table 3 Exploratory analysis of pacing parameters based on the number of diseased vessels in patients with stable CAD who underwent LBBaP.**

|  | Stable CAD | | |  |
| --- | --- | --- | --- | --- |
|  | Single vessel disease (n = 82) | | Multivessel disease (n = 22) | *P value* |
| **Intraprocedural measurements** |  |  | |  |
| Pacing threshold, V/0.4 ms | 0.69 ± 0.24 | 0.76 ± 0.44 | | 0.349 |
| R wave amplitude, mv | 11.9 ± 3.8 | 10.5 ± 3.5 | | 0.104 |
| Impedance, Ω | 757.9 ± 200.3 | 819.8 ± 258.7 | | 0.213 |
| **6-month follow-up** |  |  | |  |
| Pacing threshold, V/0.4 ms | 0.72 ± 0.24 | 0.78 ± 0.37 | | 0.336 |
| R wave amplitude, mV | 13.4 ± 4.4 | 12.5 ± 4.6 | | 0.403 |
| Impedance, Ω | 629.4 ± 130.0 | 683.4 ± 209.2 | | 0.126 |
| **12-month follow-up** | **N = 63** | **N = 20** | |  |
| Pacing threshold, V/0.4 ms | 0.73 ± 0.25 | 0.82 ± 0.37 | | 0.132 |
| R wave amplitude, mV | 13.4 ± 4.7 | 13.8 ± 4.8 | | 0.722 |
| Impedance, Ω | 627.6 ± 119.5 | 667.7 ± 188.0 | | 0.211 |

CAD: coronary artery disease; LBBaP: left bundle branch area pacing.

**Supplementary Table 4 Echocardiographic evaluation of patients with Non-CAD following LBBaP implantation, stratified by the presence or absence of LBBB.**

|  | LBBB (n = 13) | | | Without LBBB (n = 142) | | |
| --- | --- | --- | --- | --- | --- | --- |
|  | Baseline | 12-month | P value | Baseline | 12-month | P value |
| **Echocardiography** |  |  |  |  |  |  |
| LVEF, % | 41.0 ± 5.3 | 59.9 ± 9.4 | ***<0.001***** | 58.9 ± 11.4 | 61.0 ± 9.6 | 0.100 |
| LVEDD, mm | 57.5 ± 4.3 | 47.8 ± 7.2 | ***<0.001***** | 48.8 ± 8.1 | 47.2 ± 7.3 | 0.082 |

CAD: coronary artery disease; LBBaP: left bundle branch area pacing; LBBB: left bundle branch block; LVEF: left ventricular ejection fraction; LVEDD: left ventricular end-diastolic dimension; *: P < 0.05; **:P < 0.001.

**Supplementary Table 5 Echocardiographic evaluation of patients with stable CAD following LBBaP implantation, stratified by the presence or absence of LBBB.**

|  | LBBB (n = 12) | | | Without LBBB (n = 64) | | |
| --- | --- | --- | --- | --- | --- | --- |
|  | Baseline | 12-month | P value | Baseline | 12-month | P value |
| **Echocardiography** |  |  |  |  |  |  |
| LVEF, % | 40.5 ± 4.6 | 57.0 ± 7.0 | ***<0.001***** | 59.1 ± 9.8 | 60.6 ± 10.2 | 0.393 |
| LVEDD, mm | 56.7 ± 7.6 | 49.8 ± 8.0 | ***0.040**** | 49.5 ± 8.0 | 47.2 ± 7.0 | 0.080 |

CAD: coronary artery disease; LBBaP: left bundle branch area pacing; LBBB: left bundle branch block; LVEF: left ventricular ejection fraction; LVEDD: left ventricular end-diastolic dimension; *: P < 0.05; **:P < 0.001.

**Supplementary Table 6 Baseline characteristics among LBBaP, HBP, and RVP groups combined with stable CAD.**

| **Variables** | **Overall (N = 321)** | **LBBaP (N = 104)** | **HBP (N = 64)** | **RVP (N = 153)** | ***P1*** | ***P2*** | ***P3*** |
| --- | --- | --- | --- | --- | --- | --- | --- |
| Age, yrs | 70.4 ± 8.6 | 70.9 ± 7.8 | 70.4 ± 10.9 | 70.1 ± 8.1 | 0.735 | 0.451 | 0.777 |
| Male, n (%) | 185 (57.6) | 58 (55.8) | 33 (51.6) | 94 (61.4) | 0.595 | 0.364 | 0.178 |
| Hypertension, n (%) | 182 (56.7) | 65 (62.5) | 42 (65.6) | 75 (49.0) | 0.683 | ***0.033**** | ***0.025**** |
| Diabetes, n (%) | 89 (27.7) | 32 (30.8) | 15 (23.4) | 42 (27.5) | 0.304 | 0.564 | 0.540 |
| AF, n (%) | 82 (25.5) | 29 (27.9) | 14 (21.9) | 39 (25.5) | 0.386 | 0.669 | 0.572 |
| AVB, n (%) | 152 (47.4) | 50 (48.1) | 31 (48.4) | 71 (46.4) | 0.964 | 0.792 | 0.784 |
| SSS, n (%) | 134 (41.7) | 42 (40.4) | 27 (42.2) | 65 (42.5) | 0.818 | 0.738 | 0.968 |
| DCM, n (%) | 14 (4.4) | 6 (5.8) | 2 (3.1) | 6 (3.9) | 0.352 | 0.344 | 0.564 |
| HCM, n (%) | 11 (3.4) | 3 (2.9) | 2 (3.1) | 6 (3.9) | 0.632 | 0.470 | 0.564 |
| Preimplant QRS duration, ms | 110.0 ± 29.7 | 110.8 ± 32.4 | 111.1 ± 30.9 | 108.9 ± 27.2 | 0.952 | 0.627 | 0.632 |
| Multivessel disease, n (%) | 69 (21.5) | 22 (21.2) | 12 (18.8) | 32 (20.9) | 0.707 | 0.963 | 0.718 |
| cTnI, ng/ml | 0.015 (0.006, 0.036) | 0.011 (0.005, 0.032) | 0.016 (0.004, 0.035) | 0.025 (0.007, 0.041) | 0.842 | 0.165 | 0.289 |
| NT-proBNP, pg/ml | 684.0 (229.0, 1549.5) | 724.5 (276.0, 1836.5) | 797.0 (299.5, 1457.8) | 642.0 (187.5, 1573.0) | 0.669 | 0.088 | 0.444 |
| LVEF, % | 58.5 ± 11.1 | 57.4 ± 11.3 | 60.2 ± 10.0 | 58.6 ± 11.3 | 0.104 | 0.399 | 0.310 |
| LVEDD, mm | 48.4 ± 8.4 | 49.4 ± 8.5 | 47.2 ± 7.9 | 48.3 ± 8.5 | 0.090 | 0.281 | 0.373 |
| IVS thickness, mm | 10.8 ± 1.9 | 11.1 ± 2.1 | 10.5 ± 2.0 | 10.7 ± 1.8 | 0.074 | 0.112 | 0.581 |

LBBaP: Left bundle branch area pacing; HBP: his-bundle pacing; RVP: right ventricular pacing; CAD: coronary artery disease; AF: atrial fibrillation; AVB: atrioventricular block; SSS: sick sinus syndrome; DCM: dilated cardiomyopathy; HCM: hypertrophic cardiomyopathy; cTnI: cardiac troponin I; NT-proBNP: N-terminal pro-brain natriuretic peptide; LVEF: left ventricular ejection fraction; LVEDD: left ventricular end-diastolic dimension; IVS: interventricular septum; P1: LBBaP vs. HBP; P2: LBBaP vs. RVP; P3: HBP vs. RVP; *: P < 0.05.

**Supplementary Table 7 Evaluation of the safety and prognosis in stable CAD patients under the pacing modalities of LBBP and LVSP.**

|  | Stable CAD | | |  |
| --- | --- | --- | --- | --- |
|  | LBBP (n = 70) | | LVSP (n = 34) | *P value* |
| **Primary composite outcome** | 4 (5.7) | 3 (8.8) | | 0.555 |
| HFH, n (%) | 3 (4.3) | 2 (5.9) | | 0.722 |
| Upgrade to BVP, n (%) | 0 (0.0) | 0 (0.0) | | 1.000 |
| Death due to CVD, n (%) | 1 (1.4) | 1 (2.9) | | 0.600 |
| **Procedure-related complications** | 5 (7.1) | 3 (8.8) | | 0.764 |
| Septal perforation, n (%) | 0 (0.0) | 0 (0.0) | | 1.000 |
| Lead revision, n (%) | 1 (1.4) | 0 (0.0) | | 0.486 |
| Pocket hematoma, n (%) | 3 (4.3) | 2 (5.9) | | 0.722 |
| Pocket infection, n (%) | 1 (1.4) | 0 (0.0) | | 0.486 |
| Pericardial effusion, n (%) | 0 (0.0) | 1 (2.9) | | 0.151 |

CAD: coronary artery disease; LBBP: left bundle branch pacing; LVSP: left ventricular septal pacing; HFH: heart failure hospitalization; BVP: biventricular pacing; CVD: cardiovascular disease.
